# Protein aggregation with poly(vinyl) alcohol surfactant reduces double emulsion-encapsulated mammalian cell-free expression

**DOI:** 10.1371/journal.pone.0174689

**Published:** 2017-03-30

**Authors:** Kenneth K. Y. Ho, Jin Woo Lee, Grégory Durand, Sagardip Majumder, Allen P. Liu

**Affiliations:** 1 Department of Mechanical Engineering, University of Michigan, Ann Arbor, Michigan, United States of America; 2 Institut des Biomolécules Max Mousseron, UMR 5247, CNRS-Université Montpellier-ENSCM et Université d'Avignon et des Pays de Vaucluse, Avignon, France; 3 Department of Biomedical Engineering, University of Michigan, Ann Arbor, Michigan, United States of America; 4 Cellular and Molecular Biology Program, University of Michigan, Ann Arbor, Michigan, United States of America; 5 Biophysics Program, University of Michigan, Ann Arbor, Michigan, United States of America; Duke University, UNITED STATES

## Abstract

Development of artificial cell models requires encapsulation of biomolecules within membrane-bound compartments. There have been limited studies of using mammalian cell-free expression (CFE) system as the ‘cytosol’ of artificial cells. We exploit glass capillary droplet microfluidics for the encapsulation of mammalian CFE within double emulsion templated vesicles. The complexity of the physicochemical properties of HeLa cell-free lysate poses a challenge compared with encapsulating simple buffer solutions. In particular, we discovered the formation of aggregates in double emulsion templated vesicles encapsulating mammalian HeLa CFE, but not with bacterial CFE. The aggregates did not arise from insolubility of the proteins made from CFE nor due to the interaction of mammalian CFE with the organic solvents in the middle phase of the double emulsions. We found that aggregation is dependent on the concentration of poly(vinyl) alcohol (PVA) surfactant, a critical double emulsion-stabilizing surfactant, and the lysate concentration in mammalian CFE. Despite vesicle instability and reduced protein expression, we demonstrate protein expression by encapsulating mammalian CFE system. Using mass spectrometry and Western blot, we identified and verified that actin is one of the proteins inside the mammalian CFE that aggregated with PVA surfactant. Our work establishes a baseline description of mammalian CFE system encapsulated in double emulsion templated vesicles as a platform for building artificial cells.

## Introduction

Artificial cells are cell-like entities that mimic certain properties or functions of natural cells.[1, 2] Building artificial cells, a natural extension of biochemical reconstitution, provides a complimentary approach to reductionist cell biology to uncover cellular design principles.[3] Construction of artificial cells has two important criteria, encapsulation of molecules by a protective membrane and a source of biomolecules as workhorse of the artificial cells. Encapsulation of proteins inside lipid vesicles or polymersomes by self-assembly is a highly inefficient encapsulation process. Electroformation and lipid hydration are common techniques for generating giant vesicles, but these techniques either are incompatible with ionic solutions or have low encapsulation efficiency and uncontrollable vesicle lamellarity.[4, 5] A variety of microfluidic approaches can overcome these limitations and provide a facile way to encapsulate and compartmentalize biomolecules.[6–8] A particularly attractive ‘cytosol’ of artificial cells is cell-free expression (CFE) system due to its ability to synthesize any proteins of interest.[9] Our CFE system contains a complex solution largely consisting of cell-free lysate and a variety of small molecule supplements as energy sources and metabolites. These complex machineries allow for transcription and translation of desired proteins. While microfluidic encapsulation of protein solutions in simple buffers in lipid vesicles or polymersomes is robust, significantly less is known about vesicle encapsulation of CFE systems.

For both applied and basic science, in particular for the creation of artificial cell-like systems, there are considerable interests in encapsulation of biomolecules inside biomimetic membrane within diameters of 10–100 μm. Lipid membrane is a natural and deformable substrate for lipid-protein interactions that can interact with cytoskeletal proteins.[10, 11] However, lipid vesicles tend to be fragile and difficult to produce and robust encapsulation of CFE is a formidable challenge facing bottom-up assembly of artificial cells. One approach in forming lipid vesicles is the use of water-in-oil-in-water double emulsions as templates.[12] When combined with microfluidics, this approach offers the advantages of high encapsulation efficiencies with controlled compartment sizes. Typically, phospholipids are dissolved in organic solvents, which are then evaporated over time to form the membrane of the vesicles. Stable lipid vesicle formation requires slow solvent evaporation.[12] To shorten the evaporation time, thin double emulsions with thin middle oil phase were used as a template to form lipid vesicles.[13] Because of the use of organic solvents, glass capillary microfluidic devices have been used instead of more conventional polydimethylsiloxane (PDMS)-based microfluidics. However, recent advance in coating PDMS microchannels with a glass-like layer has circumvented the incompatibility problem of organic solvents with PDMS,[14] and PDMS-based microfluidic platform is now suitable for making liposomes.[7, 15] Another approach based on microfluidic jetting uses a droplet interface bilayer where a pulse of fluid to be encapsulated is jetted at high speed against the bilayer to form a vesicle in a serial manner.[16, 17] Although asymmetric lipid bilayer vesicles can be made that is unique to using this approach,[18] the entrainment of fluid during microfluidic jetting would dilute the encapsulated solutions. To date, double emulsion template is still the most reliable, high throughput, controlled, and flexible vesicle generation method available.

Cell-free protein synthesis holds tremendous promise as the cytosol of artificial cells. Development of CFE has been driven by a growing demand for an efficient and simple protein production method.[19] As a cell-free system where cell viability and survival does not need to be considered, CFE only produces protein(s) of interest. *E*. *coli* has been the predominant source of cell extracts, and integration of biological parts is conceivable as an approach towards the construction of artificial cells.[2, 20] Vesicles containing bacterial CFE as an artificial cell platform have been demonstrated in the earlier days using a reverse emulsion technique,[21] where the yield and vesicle sizes can be highly heterogeneous. Microfluidic encapsulation of bacterial CFE by double emulsion template was only achieved recently.[22, 23] As the development of CFE matures and its use becomes more widespread, there is more interest in the use of mammalian CFE. For the production of proteins of eukaryotic origins with proper post-translational modifications, mammalian CFE is more advantageous over bacterial CFE. HeLa cell-derived *in vitro* coupled transcription/translation system with supplemented transcription and translation factors has been developed.[24, 25] Using this mammalian CFE system, our group has recently demonstrated a method to encapsulate CFE inside the double emulsion templated vesicles and showed the expression of both soluble and membrane proteins, but vesicle yield was low. [26] However, to our knowledge, a systematic investigation of how solvents and surfactants required for encapsulation of mammalian CFE by double emulsion template affect the CFE expression has never been reported. In particular, since mammalian HeLa cell lysate is a highly complex and highly concentrated solution, how its expression level will be influenced by the encapsulated environment is not entirely well understood.

In this paper, we report the observation of macroscopic aggregates when mammalian CFE and double emulsion-stabilizing surfactant poly(vinyl) alcohol (PVA) were encapsulated inside double emulsion templated vesicles. The aggregates did not appear to be caused by the insolubility of membrane proteins synthesized by the CFE nor the organic solvents used as the middle phase of the double emulsions to dissolve phospholipids. We found that the aggregates were caused by high protein concentration in the mammalian HeLa CFE and high PVA surfactant concentration required for the formation of double emulsion templated vesicles. Interestingly, these aggregates were not found in encapsulated bacterial CFE system, which has a lower total protein concentration. Although protein production is reduced at the concentration of PVA used that is necessary for double emulsion droplet formation, the appearance of aggregates does not completely abrogate protein synthesis. Using mechanosensitive channel of large conductance (MscL) as a model test case, we produced double emulsion templated vesicles expressing MscL from encapsulated mammalian CFE. In an attempt to identify the proteins that aggregated with PVA surfactant, we found that actin is one of the proteins that aggregated with PVA surfactant. By systematically examining different double emulsion droplet generation conditions, our work establishes a baseline description of mammalian CFE system encapsulated in double emulsion templated vesicles as a platform for building artificial cells.

## Experimental section

### Materials

Lipids used for the formation of lipid vesicles were purchased from Avanti Polar Lipids (DOPC: 850375; DPPC: 850355; cholesterol: 700000; 16:0 Liss Rhod PE: 810158; PEG550-PE: 880530). Organic solvents, including chloroform and toluene, were purchased from Sigma-Aldrich. Hexane was purchased from Fisher Scientific. We used different types of surfactant in the study, including Triton X-100 (Sigma-Aldrich, X-100), Pluronic F-68 (Thermo Fisher, 24040032), Pluronic F-88 (BASF, 560840), Pluronic F-127 (Sigma-Aldrich, P2443) and poly(vinyl alcohol) (MW: 13,000–23,000; 87–89% hydrolyzed) (PVA) (Sigma-Aldrich, 363170). Fluorinated surfactant, F6-TAC (degree of polymerization ~ 10, MW ~ 2100 g/mol) and F8-TAC (degree of polymerization ~ 10, MW ~ 2200 g/mol), was synthesized according to previously published procedures.[27] Streptolysin-O used for the dye influx assay was purchased from Sigma-Aldrich (S5265).

### Mammalian cell-free expression

**Fig 1:**
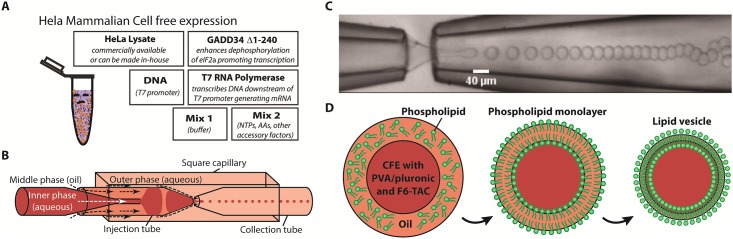
Encapsulation of Cell-Free Expression (CFE) and formation of lipid vesicles. (A) HeLa mammalian CFE system is a combination of HeLa lysate, Mix 1 buffer, GADD34, T7 RNA polymerase, Mix 2 accessory factors and DNA plasmid containing T7 promoter. (B) Thin double emulsions are formed in the glass capillary microfluidic device. (C) Image of thin double emulsion formation using the glass capillary microfluidic device. (D) Lipid vesicles are formed by first forming aqueous-in-oil-in-aqueous double emulsions where phospholipids are dissolved in volatile middle phase organic solvents. CFE system was encapsulated with surfactant (PVA/pluronic) and fluorinated surfactant (F6-TAC/F8-TAC) inside the lipid vesicles. Phospholipids self-assemble to form phospholipid monolayers on the aqueous-oil and oil-aqueous interfaces. Upon solvent evaporation, lipid vesicles form.

The mammalian CFE is a mixture of six different solutions (Fig 1A), each described below. HeLa lysate was prepared according to previously published procedures.[26] HeLa cells were obtained from Dr. Edgar Meyhofer (University of Michigan) who acquired the cells from ATCC. DNA constructs encoding GADD34 and T7 RNA polymerase were obtained from H. Imataka (University of Hyogo, Japan) and purified following previously established protocols.[24, 25] Mix 1 has a composition of 27.6 mM Mg(OAc)_2_ and 168 mM K-Hepes (pH 7.5). Mix 2 has a composition of 12.5 mM ATP, 8.36 mM GTP, 8.36 mM CTP, 8.36 mM UTP, 200 mM creatine phosphate, 7.8 mM K-Hepes (pH 7.5), 0.6 mg/mL creatine kinase, 0.3 mM amino acid mixture, 5 mM spermidine and 44.4 mM DTT. MscL-eGFP was cloned into pT7CFE vector (ThermoFisher). Mammalian CFE reactions were prepared by first mixing 9 μL lysate, 2.25 μL Mix 1 and 2.7 μL GADD34 (to give a final concentration of 310 nM) and incubated at 32°C for 10 min. Then, 2.25 μL Mix 2, 1.8 μL T7 RNA polymerase (to give a final concentration of 450 nM) and 1.5 μL DNA (500 ng) were added to form the complete CFE reaction and incubated at 32°C for 5 hr to synthesize proteins of interest. Larger total volume was also used for the encapsulation in lipid vesicles while all the volume ratios remained the same. For the bulk condition, 15 μL of HeLa CFE reaction with or without dilution at different concentrations of PVA was added to Nunc 384 well-plate and incubated at 32°C. The fluorescence intensity of the HeLa CFE reaction was measured over time in a fluorescence plate reader (BioTek Instruments, Synergy H1 Multi-Mode Reader). For single emulsion generation, 5 μL of HeLa CFE reaction was added to 100 μL of 2% Span 80 in mineral oil and vortexed for 10 seconds to generate lysate-containing single emulsions of various sizes. Unpaired t-test was used for all statistical analysis.

### Droplet microfluidic device and double emulsion templated cesicle generation

Glass capillary droplet microfluidic device was used for thin double emulsion formation (Fig 1B). The device was fabricated by assembling tapered round capillary, square capillary and syringe needles together using 5 Minutes Epoxy (Devcon, 14250).[26, 28] First, a round capillary (Sutter Instrument B100-58-10) was pulled using a pipette puller (Sutter Instruments P-87) into a tapered shape. The inner diameter of two tapered capillaries were modified using 1200 grade sandpaper to obtain inner diameters of around 50 and 80 μm for the injection tube and collection tube respectively. The inner diameters of the two tapered capillaries can vary to achieve double emulsions of different sizes. The inside surface of the injection tube was treated with trichloro(1H,1H,2H,2H-perfluorooctyl)silane to render the surface hydrophobic. Then, the injection tube and collection tube were inserted into a square capillary (AIT, 810–9917) placed on a glass slide and were aligned using an optical microscope. Another round capillary was pulled above a fire flame to form two long and thin pipettes to obtain an outer diameter of around 200 μm. One pulled pipette was inserted into the back of the injection tube and aligned using the optical microscope. Syringe needles (McMaster Carr, 75165A677) were cut and glued to the glass slide using 5 Minutes Epoxy. At last, microtubings (Scientific Commodities, BB31695-PE/5) were connected to the syringe needles.

Three syringe pumps (New Era Pump Systems, NE-500) were used to pump inner, middle and outer solutions into the glass capillary droplet microfluidic device for the formation of double emulsions. The inner solution consisted of the mammalian CFE with 2% surfactant (PVA or Pluronic surfactant) and fluorinated surfactant (2 mM of F6-TAC or 0.6 mM of F8-TAC). The middle phase was a mixture of chloroform/toluene or chloroform/hexane at a ratio of 36:64 or 40:60 (vol/vol). 9mM lipid with compositions of 69.5% DOPC, 30% cholesterol and 0.5% 16:0 Liss Rhod PE or 34.8% DOPC, 34.7% DPPC, 30% cholesterol and 0.5% 16:0 Liss Rhod PE were dissolved in the solvents. The outer solution composed of 10% PVA with 20 mM K-HEPES (pH 7.5), 150 mM KCl and glucose. The osmolarities of the inner and outer solutions were measured by using 5600 Vapro Vapor Pressure Osmometer (ELITechGroup) and the osmolarity of the outer solution was matched with that of the inner solution (~670 mOsm) by adding glucose. Flow rates of 200 μL/h, 200 μL/h and 2400 μL/h were used for the inner, middle and outer phases respectively. Fig 1C shows the formation of thin double emulsions in the glass capillary microfluidic device. Once the inner solution was encapsulated inside the double emulsions, phospholipids self-assemble to form monolayers on the aqueous-oil and oil-aqueous interfaces. The double emulsions were kept at a closed environment with slow evaporation to form lipid vesicles (Fig 1D).

### Microscopy

Thin double emulsion generation was imaged using a high-speed camera (Phantom Miro eX2) at 3000 fps on an Olympus inverted microscope. Other brightfield or fluorescence images were obtained on a Nikon inverted microscope (TiE) or on an Olympus spinning disk confocal microscope (IX73 with Yokogawa CSU X1).

### Protein identification by LC-tandem mass spectrometry

HeLa CFE were incubated at 32°C for 5 hour with different PVA concentrations. The samples were then centrifuged at 16,100 g for 10 minutes at 32°C to separate the supernatant and the pellet. The supernatant was mixed with 4x SDS sample buffer while the pellet was mixed with 1X SDS sample buffer (same final SDS concentration) and both samples were denatured for 5 min at 95°C. Samples were run on SDS-PAGE and stained with SimplyBlue SafeStain (Thermo Fisher Scientific). The protein bands of interest were excised and submitted to the Proteomics Resource Facility at the University of Michigan for protein identification using LC-MS based approach according to established protocol. Briefly, gel slices were destained with 30% methanol for 4 h. Upon reduction (10 mM DTT) and alklylation (65 mM 2-chloroacetamide) of the cysteines, proteins were digested overnight with 250 ng of sequencing grade, modified trypsin (Promega). Resulting peptides were resolved on a nano-capillary reverse phase column (Acclaim PepMap C18, 2 micron, 50 cm, ThermoScientific) using 0.1% formic acid/acetonitrile gradient at 300 nl/min (2–30% acetonitrile in 60 min followed by a 90% acetonitrile wash for 5 min and a further 25 min re-equilibration with 2% acetonitrile) and directly introduced in to Q Exactive HF mass spectrometer (Thermo Scientific, San Jose CA). MS1 scans were acquired at 120K resolution. Data-dependent high-energy C-trap dissociation MS/MS spectra were acquired with top speed option (3 sec) following each MS1 scan (relative CE ~28%). Proteins were identified by searching the data against Homo sapiens database (UniProtKB) using Proteome Discoverer (v2.1, Thermo Scientific). Search parameters included MS1 mass tolerance of 10 ppm and fragment tolerance of 0.1 Da; two missed cleavages were allowed; carbamidimethylation of cysteine was considered fixed modification and oxidation of methionine, deamidation of asparagine and glutamine were considered as potential modifications. False discovery rate (FDR) was determined using target-decoy strategy and proteins/peptides with a FDR of ≤1% were retained.

### Protein identification by Western blot

Sample preparation for Western blot was identical as described in the previous section. Samples were separated by SDS-PAGE and transferred to a nitrocellulose membrane. The membrane was washed, blocked by 5% milk, and probed using anti-actin antibody (Santa Cruz Biotechnology sc-8432). Three independent experiments were performed to confirm the obtained result. Intensity of gel bands was quantified using Image Studio Lite software.

## Results and discussion

### Aggregate formed in CFE encapsulated vesicles

**Fig 2:**
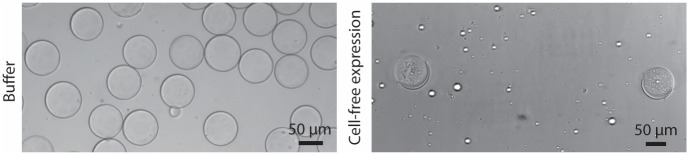
Aggregate was observed when CFE is encapsulated in the vesicle. (Top) Buffer solution (20 mM HEPES pH 7.5, 400 mM sucrose, 2% PVA, 8% PEG) was encapsulated as the inner phase using capillary droplet microfluidics with DOPC/cholesterol dissolved in chloroform/hexane as the middle phase (see materials and methods) (Bottom) HeLa CFE (18 μl HeLa lysate, 4.5 μl Mix 1 buffer, 5.4 μl GADD34, 4.5 μl T7 RNA polymerase, 3.6 μl Mix 2 accessory factors and 3 μl MscL DNA plasmid containing T7 promoter) with 2% PVA was encapsulated with DOPC/cholesterol dissolved in chloroform/hexane as the middle phase and incubated at 32 degrees in a closed environment.

Since HeLa cell lysate is a highly complex and highly concentrated solution, it is essential to define experimental conditions within a vast parameter space where mammalian CFE is compatible with microfluidic double emulsion generation. With a long term goal to develop mammalian CFE for expressing a variety of proteins (including membrane proteins) within artificial cells, it is critical to first ensure the stable self-assembly of lipid bilayer membrane. As a starting point, using capillary droplet microfluidics, we first produced double emulsion templated vesicles at a high yield encapsulating 2% PVA surfactant solution (Fig 2, top). To demonstrate the unilamellarity of the double emulsion templated vesicle, we performed membrane permeability test using a membrane pore protein streptolysin-O (S1A Fig) and showed membrane phase separation using ternary lipid mixtures (S1B Fig). Recognizing that CFE of membrane proteins can be more facile and efficient than conventional biochemical purification,[29, 30] we use MscL as a test case for membrane protein incorporation in double emulsion templated vesicles. Interestingly, we observed large aggregation inside double emulsion templated vesicles that were visible under brightfield microscopy when mammalian CFE was encapsulated (Fig 2, bottom).

### Aggregate formation was not due to insoluble membrane protein or organic solvent

**Fig 3:**
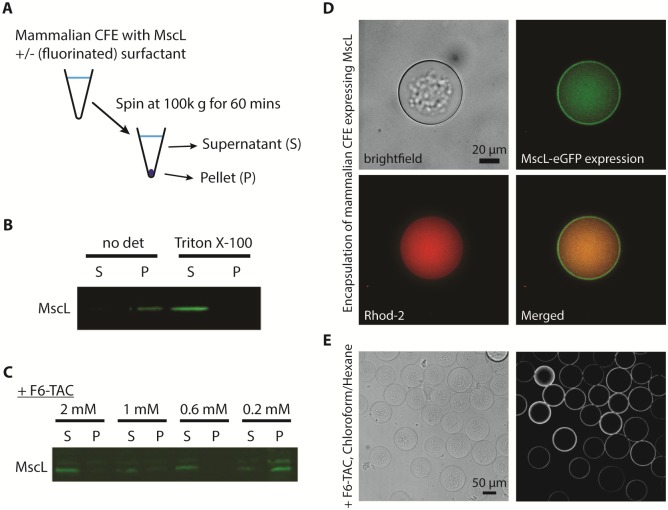
CFE of MscL is solubilized in fluorinated surfactants. (A) Schematic illustration of the solubility assay for CFE of MscL. (B) Solubility of CFE of MscL after 5 hr in the presence or absence of 0.2% of Triton X-100. (C) Solubility of CFE of MscL after 5 hr at different concentrations of fluorinated surfactant F6-TAC. (D) Formation of vesicles encapsulating mammalian CFE expressing MscL in the presence of 2% PVA and 0.6 mM F8-TAC. The vesicles were formed from 36/64 chloroform/hexane as the middle phase, and imaged in brightfield (top left) and in green fluorescence (top right). 5 μM of calcium indicator Rhod-5N and 1 mM of calcium was also encapsulated inside the vesicle (bottom left). Merged image of green and red fluorescence is shown in the bottom right. (E) Formation of HeLa lysate encapsulated vesicles formed from 40/60 chloroform/hexane in the presence of 2% PVA and 2 mM F6-TAC, imaged in brightfield (left) and in fluorescence (right).

We first suspected that MscL was aggregating since membrane proteins usually require detergent at a concentration above critical micelle concentration (CMC) to be solubilized in an aqueous solution. During biochemical purification of MscL, a nonionic detergent Triton X-100 is typically used to solubilize MscL. Using a centrifugation assay (Fig 3A), we found that MscL produced by CFE aggregated in the absence of detergent, but was solubilized in the presence of 0.2% Triton X-100 (Fig 3B). However, using Triton X-100 above CMC will also solubilize lipid bilayer if it were to be used during the encapsulation of CFE solutions.

To examine the possibility that the aggregate inside the vesicle was due to the aggregation of MscL, we examined the use of another class of surfactants made of a perfluorinated chain in its ability to solubilize MscL made by CFE. Fluorinated surfactants (FS) are amphiphilic compounds whose hydrophobic moiety consists of a perfluoroalkyl chain, usually linked to a polar head via a short hydrogenated spacer.[31, 32] In addition to being chemically and thermally stable, perfluorinated chains are both hydrophobic and lipophobic, which confers peculiar properties to FS. The strong hydrophobic interactions among fluorinated chains of FS result in very stable self-assemblies in aqueous solutions. F6-TAC is one of the fluorinated surfactants that has been used for membrane protein cell-free synthesis [27] or in membrane protein folding.[33] Solubility of MscL increased with F6-TAC concentration with almost no MscL found in the pellet at 2 mM F6-TAC (Fig 3C). 0.6 mM F6-TAC was the lowest concentration to yield soluble MscL. F8-TAC, the same type of fluorinated surfactants as F6-TAC but with longer fluorinated chain, also solubilized MscL at concentration higher than 0.6 mM (data not shown). Since the chaperone-like activity of F8-TAC was found to be reduced at high concentration,[33] likely due to its lower CMC value compared to F6-TAC, 2 mM F6-TAC or 0.6 mM F8-TAC were used in subsequent experiments without noticeable differences.

Despite inclusion of FS in CFE when expressing MscL, aggregation was still observed inside the double emulsion template vesicle (Fig 3D). Nonetheless, MscL-eGFP was found to associate with the vesicle membrane and the calcium indicator dye occupied exclusively the lumen of the vesicle. We next wondered if the aggregates could be due to other CFE proteins not related to MscL expression. After overnight incubation at 32 degrees in a closed environment, we still found aggregates inside CFE and FS encapsulated vesicles without MscL, as clearly evident from the brightfield images (Fig 3E). These results suggest that components other than MscL are causing aggregations in our experimental system.

We next examined the effect of middle phase solvents on the formation of aggregate inside CFE-containing double emulsion templated vesicle. Toluene and hexane mixtures with chloroform are commonly used as the middle phase solvents in the generation of double emulsion droplets containing diblock copolymers or phospholipids.[13, 34, 35] 36–40% volume percent of chloroform has been shown previously to yield favorable adhesion energy between the two stabilized monolayers, thus supporting dewetting and the formation of vesicles.[35] At higher chloroform concentrations (above 43%), double emulsion droplets become unstable. Since chloroform has higher water solubility than hexane or toluene, it will diffuse into the outer phase and evaporate. The resultant hexane/toluene-rich solvent is poor for phospholipid and help drive the attraction of two lipid monolayers in a dewetting transition.[13]

All the double emulsion droplets generated above used chloroform/hexane as the middle phase to dissolve the lipid mixture. Thin double emulsion droplets encapsulating mammalian HeLa CFE were generated using chloroform/toluene as the middle phase solvent. After overnight incubation at 32 degrees in a closed environment, we found aggregates on or inside the vesicles with a dark ring around many vesicles (S2 Fig). Interestingly, some vesicles appeared to have dewetted (‘yellow’ arrows). Occasionally, we found vesicles extruded from the aggregates, suggesting a more solid or gel-like property of this aggregates. From these experiments, we concluded that the formation of aggregate was likely not due to the insolubility of MscL or the type of organic solvents used as the middle phase.

### Aggregate formed in the presence of 2% PVA

**Fig 4:**
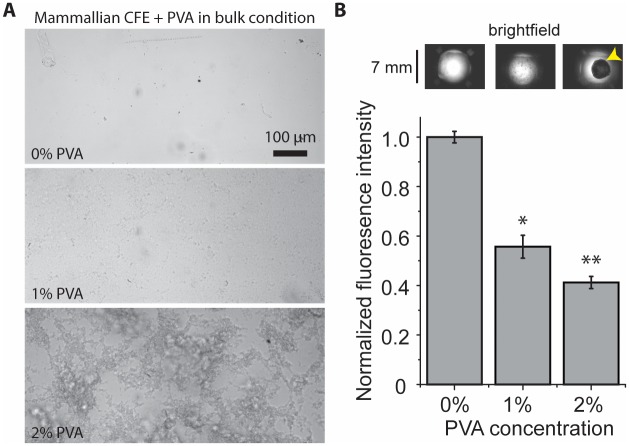
PVA surfactant caused the formation of aggregate and reduced CFE activity. (A) Brightfield images of mammalian CFE at 0, 1, or 2% of PVA surfactant. (B) eGFP expression in HeLa CFE as a function of PVA concentration measured in microwell plates (n = 3, ±S.E.), unpaired t test comparing with 0%; *: *p* < 0.01; **: *p* < 0.001. Brightfield images of microwell corresponding to the different PVA concentration are shown on top. Yellow arrowhead points to the large aggregate in the microwell.

We speculated the aggregates could be precipitation of proteins in cell lysates over the course of the experiment due to some unknown chemical reactions between the lysate and the surfactant used in double emulsion generation. Formation of thin shell double emulsion droplets consists the use of poly(vinyl alcohol) (MW: 13,000–23,000; 87–89% hydrolyzed) (PVA) at 2 wt% in the inner phase and 10 wt% in the outer phase.[13, 26] PVA of this type has been widely used as a polymeric surfactant in enhancing the stability of double emulsion droplets. To test whether the aggregates were a result of the PVA surfactant, we mixed mammalian CFE with different concentrations of PVA surfactant under bulk condition, which is a simple and facile approach for testing different conditions without the influence of middle phase organic solvents. Consistent with our findings in double emulsion templated vesicles, we observed macroscopic aggregates at 2% PVA (Fig 4A). However, these macroscopic aggregates did not appear when PVA concentration was lower than 2% (smaller aggregates were visible for 1% PVA). We further performed CFE of enhanced green fluorescence protein (eGFP), under bulk conditions, at different concentrations of PVA. Interestingly, we also observed a concentration dependent decrease of eGFP expression with PVA concentration. At 2% PVA concentration, we found the eGFP expression was reduced to ~40% compared to that without surfactant (Fig 4B). This result points to the possibility that the use of droplet stabilizing surfactant PVA plays a role in generating the macroscopic aggregates and reduces cell-free protein expression.

### Pluronic surfactant also generated aggregates

**Fig 5:**
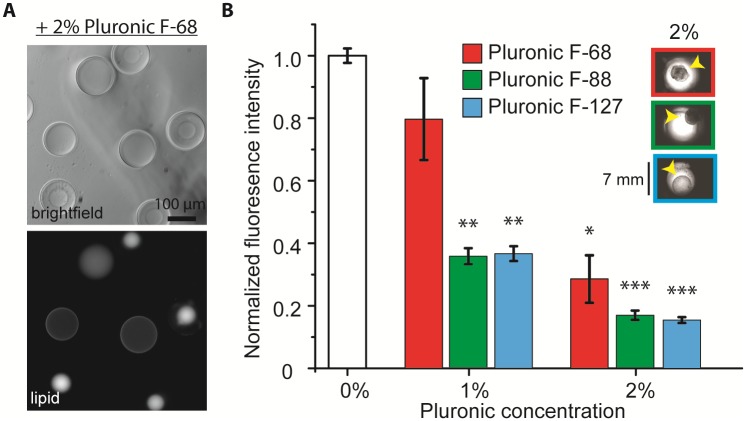
Pluronic surfactant also caused the formation of aggregate and reduced CFE activity. (A) Brightfield (top) and fluorescence (bottom) images of double emulsion templated vesicles (without HeLa lysate) with 2% Pluronic F-68. (B) eGFP expression in HeLa CFE at different concentrations of surfactants for Pluronic F-68 (red), F-88 (green), and F-127 (blue), unpaired t test comparing with 0%; *: *p* < 0.01; **: *p* < 0.001; ***: *p* < 0.0001. Inset shows brightfield microwell images at 2% of surfactant concentration with yellow arrowhead pointing to the aggregates.

Since the surfactant PVA causes aggregation in our experimental system, we next examined the propensity for aggregate formation with other biocompatible surfactants. Another common surfactant used in emulsification process and in cell culturing is block copolymers of polyoxyethylene-polyoxypropylene-polyoxyethylene (PEO-PPO-PEO, also known as Pluronic). The hydrophilicity of Pluronic is determined by the different numbers of the repeating units of PEO (relatively hydrophilic) and PPO (relatively hydrophobic). When we used Pluronic F-68 instead of PVA as the surfactant without encapsulating HeLa lysate, we could immediately observe dewetting, as indicated by the formation of a small lipid reservoir, evident in both brightfield and fluorescence images (Fig 5A). At 2% surfactant concentration, the formation of aggregates was observed with three different Pluronic surfactants, F68, F88, and F127 (Fig 5B inset). Pluronic F68, F88, F127 are widely used in cell culture and are compatible with biological components. Pluronic F88 and F127 had significant reduction in protein expression compared to Pluronic F68 (Fig 5B). The absence of middle phase solvents in these experiments again supports the idea that the interaction between surfactant and mammalian CFE alone is sufficient for the formation of these macroscopic aggregates over time. Nonetheless, CFE could still occurred at some capacity. Although we could not observe large aggregates at 1% surfactants, double emulsion droplets would fail to be produced at 1% surfactant.

### Aggregate formed under high concentration of mammalian CFE

**Fig 6:**
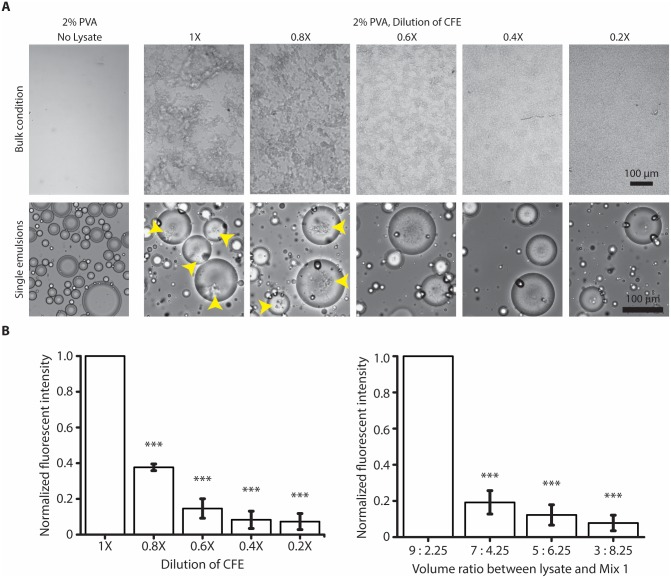
High concentration of mammalian Hela lysate is prone to aggregation when exposed to surfactant. (A) (Top) Representative brightfield images of different dilution of CFE reaction with 2% PVA under bulk condition. (Bottom) Representative brightfield images of single emulsions encapsulating 2% PVA at different CFE dilutions. Yellow arrowheads denote the appearance of aggregates. (B) (Left) eGFP expression in HeLa CFE as a function of CFE dilution measured in microwell plates (n = 4, ±S.E.), unpaired t test comparing with 1X; ***: *p* < 0.0001. (Right) eGFP expression in HeLa CFE at different volume ratios between lysate and Mix 1 measured in microwell plates (n = 4, ±S.E.), unpaired t test comparing with 9:2.25 volume ratio; ***: *p* < 0.0001.

Since having surfactants is important for stabilizing double emulsion droplet generation, we wondered if the lysate concentration could play a role in the formation of the aggregates. We tested this under bulk condition and used a single emulsion setup containing 2% PVA at different HeLa lysate dilutions to simulate the encapsulated environment. Single emulsion setup is a quick and easy way of creating an encapsulated environment without the presence of volatile organic solvents. At 100% mammalian CFE concentration (11.2 ± 1.0 mg/mL), we could find aggregates under bulk condition and in single emulsion with 2% PVA across different droplet sizes. Interestingly, we no longer observed aggregates when the CFE concentration was diluted to 40% under bulk condition or 60% in single emulsion (Fig 6A). While this result seems encouraging, we find rapidly diminishing eGFP expression with dilution of the entire CFE system (Fig 6B, left) or dilution of the HeLa lysate alone by varying the volume ratio between lysate and Mix 1 (Fig 6B, right).

In support of our discovery that the high concentration of mammalian HeLa lysate in conjunction with surfactant led to the formation of aggregates, we find no aggregates when we mixed bacterial CFE system with 2% PVA (S3A Fig) or encapsulated bacterial CFE system (6.4 ± 0.9 mg/mL) using double emulsion templated vesicles (S3B Fig, top). In particular, 2% PVA has no impact on the expression of eGFP by bacterial CFE (S3B Fig, bottom).

### Actin aggregated with PVA

**Fig 7:**
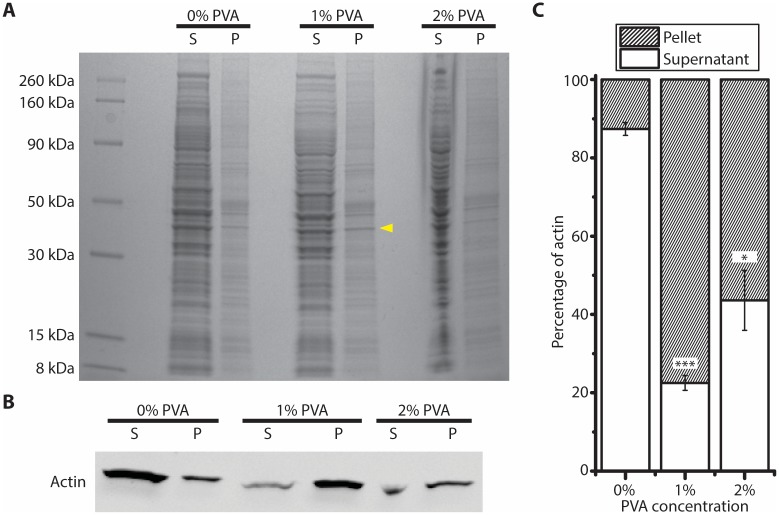
Actin aggregates with PVA surfactant. (A) Coomassie blue stained gel showing proteins in the supernatant or pellet after 10 min centrifugation at 16,100 g, in the presence of PVA surfactant at different concentrations. (B) Western blot showing the actin in the supernatant or pellet fractions at different PVA concentrations. (C) Percentage of actin measured in the supernatant and pellet fractions at different PVA concentrations (n = 3, ±S.E.), unpaired t test comparing with 0% PVA; *: *p* < 0.01; ***: *p* < 0.0001.

Thus far, we found that high concentration of mammalian HeLa lysate in conjunction with PVA surfactant led to the formation of aggregates. However, we have not identified component(s) that formed these aggregates. To further investigate this, the mixtures of mammalian CFE with different concentrations of PVA surfactant were centrifuged to separate the aggregate/pellet and the supernatant. While the overall amount of proteins in the supernatant and pellet fractions at different concentrations of PVA did not appear to change significantly (Fig 7A), we saw more protein of molecular weight around 40–45 kDa in the pellet fraction at 1% PVA. To identify the specific protein aggregating with PVA, the protein band was cut out and LC-tandem mass spectrometry was performed. Actin was identified as the major protein in the sample (mass spectrometry; 17.8% and 21.5% of peptides were actin at 1% and 2% PVA respectively for peptide count > 1). The aggregation of actin with PVA surfactant was also verified using Western blot (Fig 7B). The percentage of actin in the pellet fraction increased significantly when the mammalian CFE was incubated with 1% or 2% of PVA surfactant (Fig 7C). Interestingly, the percentage of actin in pellet at 2% PVA is lower than that at 1% PVA. We speculated that this is due to the inability of detergent to fully solubilize the proteins in the aggregate. This can also explain why we did not see a significant amount of proteins in the pellet fraction. Nevertheless, our results indicate that actin was one of the proteins that aggregated with PVA in the mammalian CFE reaction and we believe the concentrations of other proteins that are important for transcription and/or translation could also be reduced due to a similar mechanism.

## Conclusion

There is a great need to develop artificial cell systems for modeling cellular behaviors, discovering cellular design principles, and advancing drug delivery platforms.[36, 37] As a yet untapped area of encapsulating mammalian CFE system as the ‘cytosol’ of lipid membrane-enclosed artificial cells, we report a systematic study to examine the effect of PVA surfactant on mammalian CFE-containing double emulsion templated vesicles. Although we find 2% PVA surfactant reduces protein expression and led to the formation of macroscopic aggregates in mammalian HeLa CFE, 2% PVA surfactant is critical for stable microfluidic double emulsion generation. We show that the high protein concentration in HeLa CFE and PVA surfactant present a condition for these aggregates to form. We further discovered that even when the macroscopic aggregates only appeared when the concentration of PVA surfactant reached 2%, actin is one of the proteins in the mammalian CFE that aggregated with PVA. With the hydrophobic and hydrophilic parts of the PVA surfactant, it is not surprising to see that proteins important for cell-free synthesis could also be aggregating with PVA. Therefore, one needs to be cautious with using PVA and other stabilizing surfactants for double emulsions encapsulating proteins. Our work thus provides a baseline description of the current state-of-the-art in encapsulation of mammalian CFE system in double emulsion templated vesicles and offer directions for further improvement and optimization of this artificial cell system.

## Supporting information

S1 Fig Unilamellarity of the double emulsion templated vesicle membrane. (A) Dye influx assay demonstrating the insertion of a membrane-damaging protein toxin from streptococci, streptolysin O (SLO), similar to what has been shown previously.(13) The double emulsion templated vesicles contain 69.5% DOPC, 30% cholesterol, and 0.5% NBD-PE and were formed from 36/64 chloroform/hexane in the middle phase. 0.05 mg/ml of SLO was added to the outside of the vesicles and incubated with the vesicles for 1 hr at room temperature before TMR-rhodamine was added to a final concentration of ~0.12 mM. *p* < 0.05 using unpaired t test. (B) Phase separation of DOPC and DPPC at room temperature. Double emulsion templated vesicles contain 33.9% DOPC, 33.9% DPPC, 30% cholesterol, 0.2% Rhod PE, and 2% PEG-550-PE and were formed from 36/64 chloroform/hexane in the middle phase. Double emulsions were generated and collected in a closed container. The phase separated vesicles were imaged after overnight evaporation of solvents.
(TIF)

S2 Fig Aggregate was observed in the vesicle regardless of organic solvents. Formation of HeLa lysate encapsulated vesicles formed from 40/60 chloroform/toluene in the presence (top) or absence (bottom) of 2 mM F6-TAC. Yellow arrowheads denote the appearance of dewetted interface.
(TIF)

S3 Fig No aggregate formed in bacterial CFE. (A) Brightfield images of bacterial CFE without (top) and with (bottom) 2% of PVA surfactant (B) Bacterial CFE encapsulation in double emulsion templated vesicles, imaged in brightfield (top left) and in lipid fluorescence (top right). (Bottom) eGFP expression in bacterial CFE over time with and without 2% PVA measured in microwell plates.
(TIF)
