# Supplementary figures and images for: Protein aggregation with poly(vinyl) alcohol surfactant reduces double emulsion-encapsulated mammalian cell-free expression
Source: PLoS One. 2017 Mar 30;12(3):e0174689. doi: 10.1371/journal.pone.0174689 (PMC5373588; doi:10.1371/journal.pone.0174689)

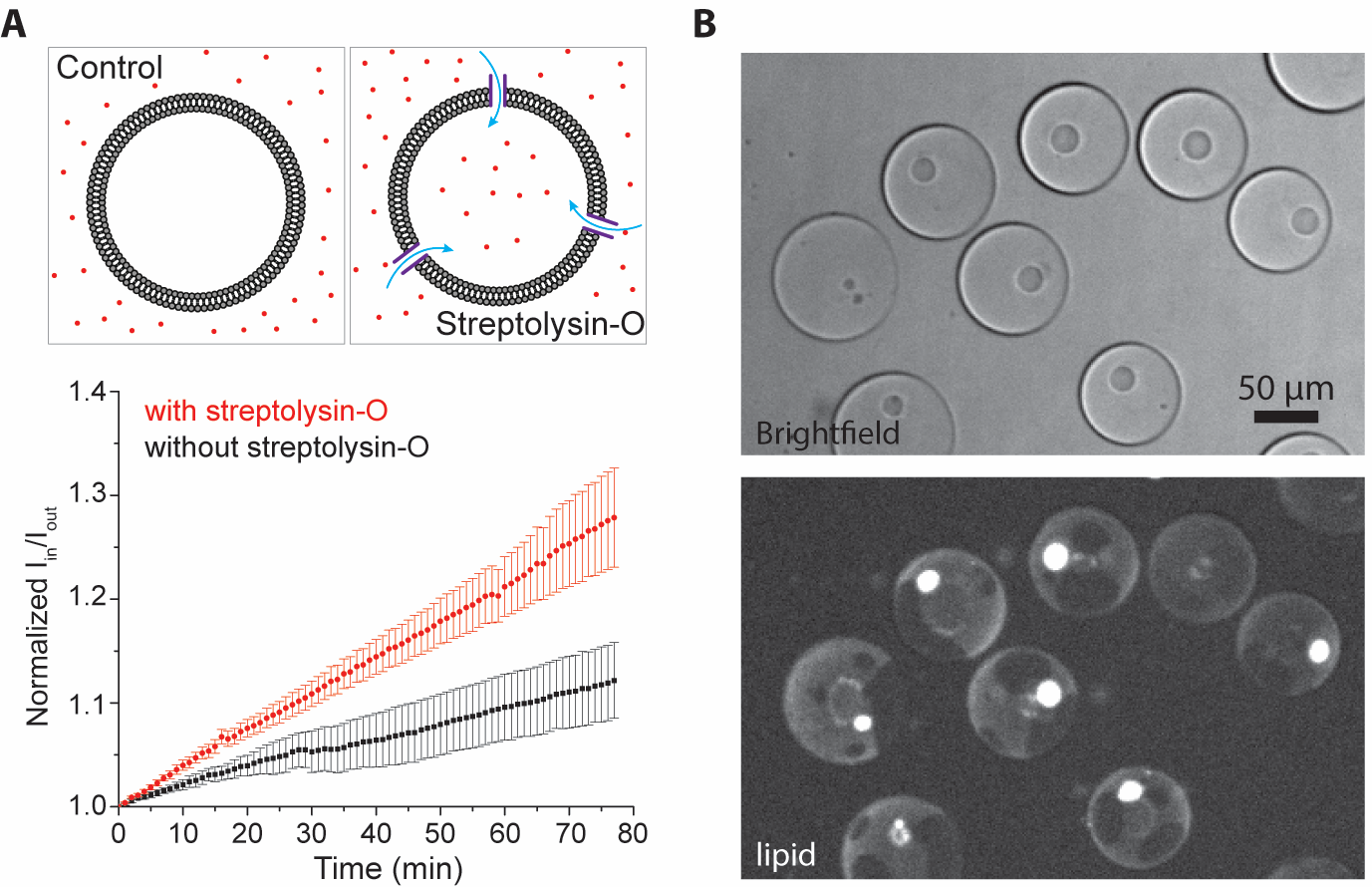

Supplement: S1 Fig — (A) Dye influx assay demonstrating the insertion of a membrane-damaging protein toxin from streptococci, streptolysin O (SLO), similar to what has been shown previously.(13) The double emulsion templated vesicles contain 69.5% DOPC, 30% cholesterol, and 0.5% NBD-PE and were formed from 36/64 chloroform/hexane in the middle phase. 0.05 mg/ml of SLO was added to the outside of the vesicles and incubated with the vesicles for 1 hr at room temperature before TMR-rhodamine was added to a final concentration of ~0.12 mM. p < 0.05 using unpaired t test. (B) Phase separation of DOPC and DPPC at room temperature. Double emulsion templated vesicles contain 33.9% DOPC, 33.9% DPPC, 30% cholesterol, 0.2% Rhod PE, and 2% PEG-550-PE and were formed from 36/64 chloroform/hexane in the middle phase. Double emulsions were generated and collected in a closed container. The phase separated vesicles were imaged after overnight evaporation of solvents. (TIF) [file pone.0174689.s001.tif]

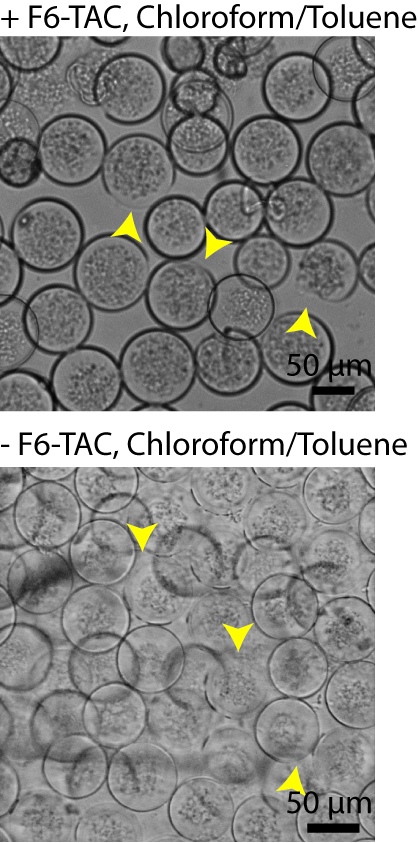

Supplement: S2 Fig — Formation of HeLa lysate encapsulated vesicles formed from 40/60 chloroform/toluene in the presence (top) or absence (bottom) of 2 mM F6-TAC. Yellow arrowheads denote the appearance of dewetted interface. (TIF) [file pone.0174689.s002.tif]

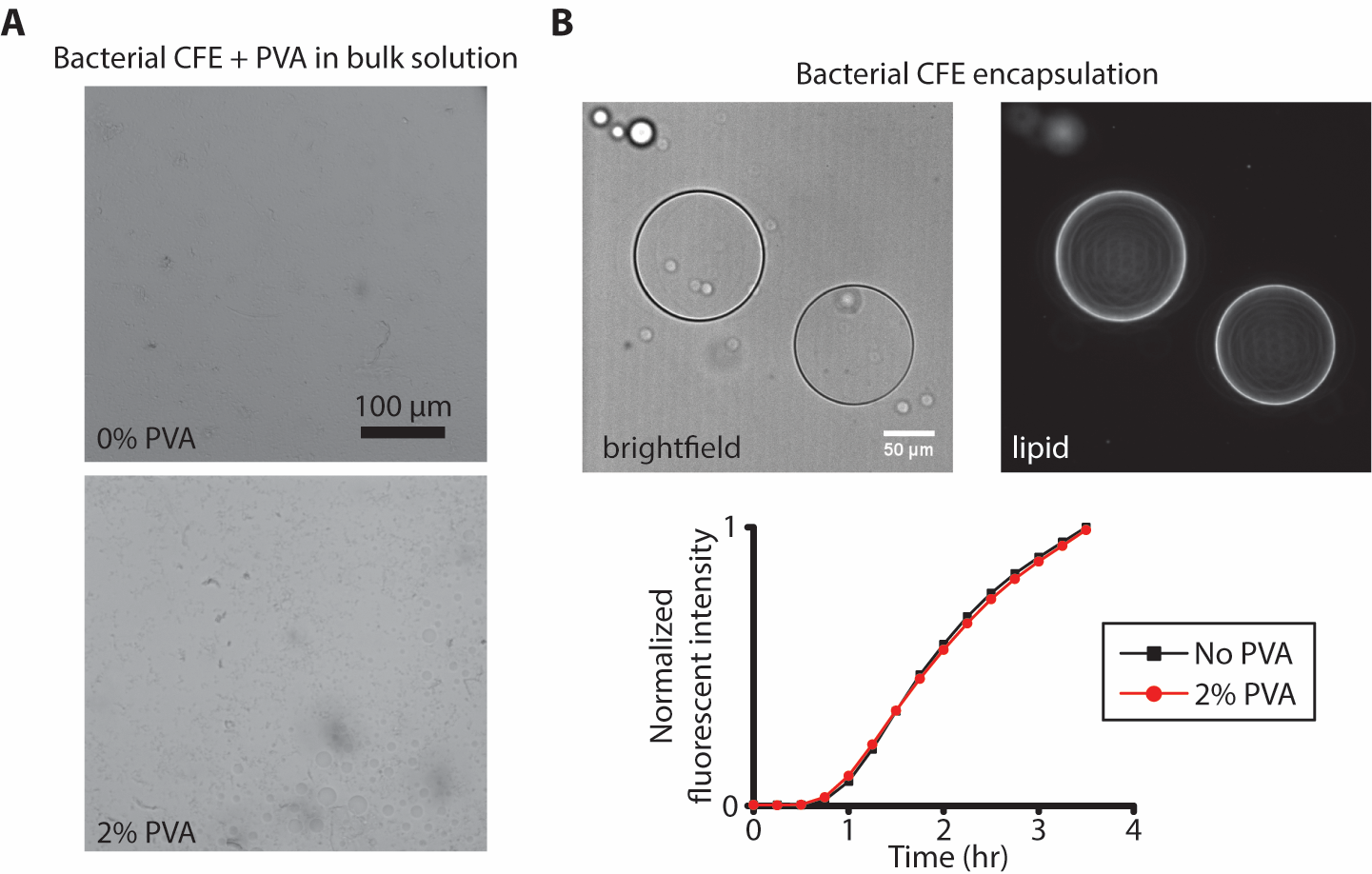

Supplement: S3 Fig — (A) Brightfield images of bacterial CFE without (top) and with (bottom) 2% of PVA surfactant (B) Bacterial CFE encapsulation in double emulsion templated vesicles, imaged in brightfield (top left) and in lipid fluorescence (top right). (Bottom) eGFP expression in bacterial CFE over time with and without 2% PVA measured in microwell plates. (TIF) [file pone.0174689.s003.tif]
